# Is There a Link between Oropharyngeal Microbiome and Schizophrenia? A Narrative Review

**DOI:** 10.3390/ijms23020846

**Published:** 2022-01-13

**Authors:** Stanislas Martin, Audrey Foulon, Wissam El Hage, Diane Dufour-Rainfray, Frédéric Denis

**Affiliations:** 1 Department of Psychiatry, Centre Hospitalier Universitaire Tours, 37000 Tours, France; sta.martin@laposte.net; 2 Faculty of Medicine, Université de Tours, 37000 Tours, France; foulon.audrey22@gmail.com; 3 U1253, iBrain, Inserm, CHU Tours, Université de Tours, 37000 Tours, France; wissam.elhage@univ-tours.fr (W.E.H.); diane.dufour@univ-tours.fr (D.D.-R.); 4 Service de Médecine Nucléaire In Vitro, Centre Hospitalier Universitaire Tours, 37044 Tours, France; 5 Department of Odontology, Centre Hospitalier Universitaire Tours, 37000 Tours, France; 6 Faculty of Dentistry, Nantes University, 44000 Nantes, France; 7 EA 75-05 Education, Ethics, Health, Faculty of Medicine, Université de Tours, 37000 Tours, France

**Keywords:** schizophrenia, oropharyngeal microbiota, oropharyngeal microbiome, neuroinflammation, dysbiosis

## Abstract

The study aimed to examine the impact of the oropharyngeal microbiome in the pathophysiology of schizophrenia and to clarify whether there might be a bidirectional link between the oral microbiota and the brain in a context of dysbiosis-related neuroinflammation. We selected nine articles including three systemic reviews with several articles from the same research team. Different themes emerged, which we grouped into 5 distinct parts concerning the oropharyngeal phageome, the oropharyngeal microbiome, the salivary microbiome and periodontal disease potentially associated with schizophrenia, and the impact of drugs on the microbiome and schizophrenia. We pointed out the presence of phageoma in patients suffering from schizophrenia and that periodontal disease reinforces the role of inflammation in the pathophysiology of schizophrenia. Moreover, saliva could be an interesting substrate to characterize the different stages of schizophrenia. However, the few studies we have on the subject are limited in scope, and some of them are the work of a single team. At this stage of knowledge, it is difficult to conclude on the existence of a bidirectional link between the brain and the oral microbiome. Future studies on the subject will clarify these questions that for the moment remain unresolved.

## 1. Introduction

Schizophrenia is a chronic serious mental illness with worldwide prevalence that affects approximately 1 in 100 people [1]. The symptoms of the condition include «positive symptoms» (e.g., delusions, hallucinations), «negative symptoms» translating an impoverishment of the psychic life (e.g., affective and emotional blunting and social withdrawal) and syndrome of disorganization (e.g., impaired language and thought processes) [2]. It usually begins between the ages of 15 and 25 [2]. Although schizophrenia is a common illness, the pathophysiology is still poorly understood. Schizophrenia is thought to be the result of an interaction between genes and environment, with genetic vulnerability being influenced by environmental factors. 

Awareness of the links between neuroinflammation and mental illness is one of the major advances of the last decade that led to the idea that chronic inflammation may have a role in the psychopathology of schizophrenia [3]. However, the pathological events leading to the observed altered inflammatory status are still poorly understood even though there is increasing and convincing evidence implicating immunological dysfunction as a key element in the pathophysiology of this mental disorder. High levels of proinflammatory cytokines and low levels of anti-inflammatory cytokines were reported in the literature [4]. For example, it was described that the blood and cerebrospinal fluid of schizophrenic patients contain high levels of proinflammatory substances, and a dose-response relationship between autoimmune diseases [5], the number of severe infections, and the risk of schizophrenia was found [6].

Recent years saw a growing interest in the potential role of the gut microbiota in mental health, notably in schizophrenia. Nikolova et al. [7] suggested a transdiagnostic commonality of microbial disturbances in certain psychiatric disorders including schizophrenia. It would be characterized by a depletion of anti-inflammatory butyrate-producing bacteria and an enrichment of proinflammatory bacteria. A link between the gut microbiota and the brain is assumed, this link is called the “gut-brain axis” (GBA). It consists of a bidirectional communication between the central nervous system and the enteric nervous system, linking the emotional and cognitive centers of the brain to the peripheral gut functions. This link appears to be bidirectional via neural, endocrine, and humoral signals between the brain and the gut microbiota. It was demonstrated by the association of dysbiosis with autism, anxiety-depressive behavior, and functional gastrointestinal disorders [8]. 

In the same vein, relationships between the oral microbiota and certain brain pathologies such as autism spectrum disorders [9] or bipolar disorders [10] appear to exist. Oral cavity is a complex environment where we can find small different microbial habitats, such as teeth, palate, mucosal, and tongue [11]. The oral microbiota is mainly composed of bacteria, viruses, fungi, protozoa, and archaea [11]. About 50 to 100 billion bacteria were identified in the oral cavity and 600 prevalent taxa at the species level [12]. These species belong to 185 genera and 12 phyla [12]. The latter include *Firmicutes*, *Fusobacteria*, *Proteobacteria*, *Actinobacteria*, *Bacteroidetes*, *Chlamydiae*, *Chloroflexi*, *Spirochaetes*, *Synergistetes*, *Saccharibacteria,* and *Gracilibacteria* [12,13]. The human oral microbiota is the second most abundant microbiota after the gastrointestinal microbiota.

Human oral microbiota has become a new research focus area, as oral bacteria, bacterial products, and inflammatory molecules can invade the human body through the bloodstream or the digestive tract. In this context, it was shown that perturbation of the oral microbiota is associated with not only infectious oral diseases but also with several systemic diseases [14], such as metabolic diseases, cardiovascular diseases, respiratory diseases, rheumatoid arthritis, inflammatory bowel diseases, Alzheimer’s disease, and autism spectrum disorders [15,16]. For instance, the pathogen in chronic periodontitis, *porphyromonas gingivalis*, was identified in the brain of Alzheimer’s disease patients and may play a role in the pathogenesis of this illness [17]. Thus, the emerging concept of “Oral-Brain-Axis” (OBA) may contribute to the understanding of the psychopathological mechanisms of psychological disorders.

Therefore, the aim of this work was to examine the impact of the oropharyngeal microbiome in the pathophysiology of schizophrenia and to clarify whether there might be a bidirectional link between the oral microbiota and the brain in a context of dysbiosis-related neuroinflammation.

## 2. Search Strategy

### 2.1. Information Sources

A systematic search of electronic databases and grey literature was conducted using the Medical Subject Headings (MeSH) terms: schizophrenia, oropharyngeal microbiota, oropharyngeal microbiome, neuroinflammation, and dysbiosis. Searches were conducted using the following search engines: MEDLINE, EMBASE, Web of Science Core Collection, and Google Scholar. In addition, PsycINFO database and Cochrane database were also searched as they are particularly relevant to the review topic of the link between oropharyngeal microbiome and schizophrenia in dysbiosis-related neuroinflammation. Search dates were not limited. To ensure saturation of the literature, we also reviewed reference lists of included studies or relevant reviews identified by the search.

### 2.2. Review and Selection

A systematic search of the databases was carried out by two independent reviewers (AF and SM). All of the identified studies were exported to a citation manager, and duplicates were removed. 

### 2.3. Data Extraction

Two reviewers (AF and SM) extracted data independently. The two authors (AF and SM) screened the title and abstract of the studies and selected the studies potentially eligible for the full text, which was assessed independently by these two authors for details. All excluded data were agreed on by both reviewers following discussion, and any disagreement was referred to a third reviewer (FD). 

## 3. Results

The articles selected from our literature search on the subject of the oropharyngeal microbiome and links with schizophrenia are summarized in Table 1.

**Figure 1:**
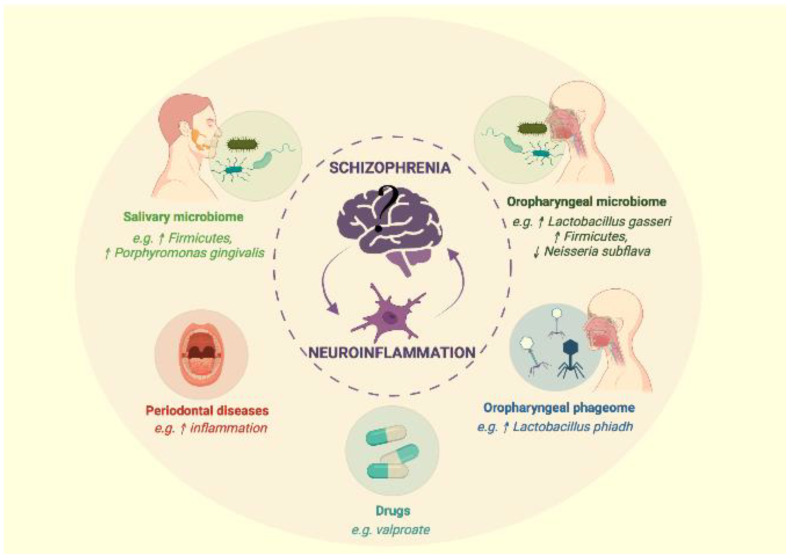
Link between oral microbiota and schizophrenia. (Figure created with Biorender.com, accessed on 25 December 2021).

We selected 9 articles including 3 systemic reviews with several articles from the same research team. In summary, different themes emerged from the analysis of these articles, which we have grouped into five distinct paragraphs concerning the oropharyngeal phageome, the oropharyngeal microbiome, the salivary microbiome, periodontal disease potentially associated with schizophrenia and the impact of drugs (such as valproate) on the microbiome and schizophrenia (Figure 1).

### 3.1. The Oropharyngeal Phageome 

Among the microorganisms that make up the microbiome, bacteriophages are viruses that infect bacteria and their metabolism and replication. There are an estimated 10^31^ phages on the planet, the most abundant life form on earth. There are two types of phages: virulent phages that cause a lytic cycle leading to the death of its host, and temperate phages that cause a lysogenic cycle, involving integration of their genome into the host chromosome to become a prophage. The host bacterium of a lysogenic phage is not destroyed and transmits this genetic material. However, under certain stress conditions the lysogenic cycle can switch to a lytic lifecycle [26]. Through metagenomic characterization of bacteriophages from the oropharynx of persons with schizophrenia (PWS), Yolken et al. [19] identified 79 distinct bacteriophage sequences. Of these, they identified only one phage significantly higher in samples from PWS regardless of age, sex, race, socioeconomic status, or smoking: *Lactobacillus phiadh,* which infects *Lactobacillus gasseri*. At least 1 match for *Lactobacillus phage phiadh* was found in 17 of 41 of PWS, as compared to 1 of 33 of controls (*p* < 0.001 Fisher’s Exact Test). The study also found that the level of *Lactobacillus phiadh* was correlated with an increased rate of comorbid immunological disorders in PWS. Thus, 9 of 17 PWS who had at least 1 match for *Lactobacillus* phage phiadh had a comorbid immunological disease (6 individuals had type 2 diabetes, 2 had type 1 diabetes, and 1 had Crohn’s disease). 

The link between schizophrenia and the phage *Lactobacillus phiadh* is not defined with certainty but it is likely that the phage *Lactobacillus phiadh* modulates the level of its host bacterium, *Lactobacillus gasseri*, which affects the host’s immune system. The phage could have several effects on its host. It could lead directly to its death or establish a long-term lysogenic state within the host bacterial genome. 

### 3.2. The Oropharyngeal Microbiome 

In the hypothesis that the oropharyngeal microbiome may be associated with or contribute to an altered immune status in schizophrenia, Castro–Nallar et al. [20] conducted a case control study to characterize the schizophrenia oropharyngeal microbiome structure regarding its taxonomic and functional diversity. Significant difference was demonstrated for the studied variables (maternal education, self-reported race, age, or gender). At the phylum level, all samples from PWS have a higher proportion of Firmicutes compared to controls where a higher relative proportion of Bacteroidetes and Actinobacteria was found. Relative proportions of other phyla such as Fusobacteria and Proteobacteria do not differ greatly between schizophrenics and the control group. In this study, smoking did not appear to affect the composition of the microbiota at the phylum level. Control group was richer in the number of species compared to that of schizophrenia sample, but dominated by fewer species, as opposed to PWS [20]. In schizophrenia, oropharyngeal samples were characterized by an increase in lactic acid bacteria (including lactobacillus and bifidobacterium), Candida, and Eubacterium together with a marked reduction of *Neisseria*, *Haemophilus*, and *Capnocytophaga* [20]. Decrease in Neisseria and Capnocytophaga abundance was associated with cigarette smoking [27]. These authors also observed an increase in the proportion of Lactobacillus gasseri, which appeared to be at least 400 times more abundant in schizophrenic patients than in controls. Castro–Nallar et al. [20] highlighted that the microbiome of PWS was characterized by an increased number of metabolic pathways related to metabolite transport systems including siderophores, glutamate, and vitamin B12. In contrast, carbohydrate, lipid pathways and energy metabolism were abundant in controls. Yolken et al. [24] found that pharyngeal microflora of 121 individuals with schizophrenia differed in composition and abundance from those of controls, as did those with other psychiatric disorders. The levels of *Neisseria subflava*, *Weeksellaceae*, and *Prevotella* were significantly lower in PWS and mania as compared to that of controls without a psychiatric diagnosis. On the other hand, levels of *Streptococci* were higher in PWS and mania, and an altered beta-diversity in PWS and mania as compared to that of control individuals without a psychiatric disorder. However, there are a number of environmental exposures that might be increased in individuals with psychiatric disorders not directly measured in this study [24]. 

### 3.3. Salivary Microbiome

The saliva is an important factor affecting oral microbiome [28]. Recently, another observational study [25] aimed to investigate the salivary microbiome in the context of schizophrenia. More specifically, it provides new data in favor of a link between salivary microbiome alterations and schizophrenia initiation. The authors identified three stages: 85 patients with first-episode schizophrenia (FES), 43 patients with a clinical high risk (CHR) and 80 healthy controls (HC). The purpose of this research was to characterize the microbial profiles at those different stages. As Yolken et al. [24], the authors confirmed the high ratio *Firmicutes/Proteobacteria* in PWS but in the salivary microbiome. The salivary of FES group has a high alpha-diversity (Shannon index) and low beta-diversity (PcoA analysis based on weighted UniFrac phylogenetic distances) heterogeneity. The two other groups (CHR and FC) were similar. Furthermore, the authors added that sulfate-reducing bacteria (or H2S-producing bacteria) could be potential diagnostic biomarkers for FES and CHR, because of the correlation between enrichment of H2S-producing bacteria in saliva and increased risk of initiation of schizophrenia. The enrichment of H2S-producing bacteria could precede the onset of the disorder and could also be linked to the clinical manifestations of schizophrenia (attenuated psychotic symptoms or first-episode schizophrenia). Finally, the study showed that the metabolic functions of the salivary microbiome were disturbed in schizophrenia, notably xenobiotic biodegradation pathways were significantly depleted in the FES group. 

### 3.4. Periodontal Diseases and Schizophrenia 

Because of the inflammatory process induced by periodontal disease, a periodontal disease approach was considered to investigate the link between oral microbiota and schizophrenia. The periodontal disease refers to infectious and inflammatory disorders that damage the soft tissue around the teeth. Periodontitis is a polymicrobial inflammatory disorder, resulting from microorganisms residing within the dental plaque, a common oral infection affiliated with gram negative, anaerobic bacteria. It elicits a “low grade systemic inflammation” by release of proinflammatory cytokines into systemic circulation [29]. The predominant periodontal pathogens involved in periodontitis are *Aggregatibacter actinomycetemcomitans*, *Porphyromonas gingivalis*, *Prevotella intermedia*, *Fusobacterium nucleatum*, *Tannerella forsythensis*, *Eikenella corrodens,* and *Treponema denticola* [29,30]. 

Systemic inflammation can lead to increased levels of inflammatory cytokines in the central nervous system associated with activation of glial cells and the immune cells in the brain, this is neuroinflammation [31]. Hashioka et al. [22] summarized how does periodontitis cause neuroinflammation. They found three possibilities: (1) cytokines produced as a result of the inflammation caused by carries can communicate with the brain via the neural pathway, humoral pathway, and cellular pathway; (2) bacteria or molecules produced by bacteria can directly invade the brain, e.g., through the bloodstream or the cranial nerves; (3) communication could take place in the leptomeninges. Neuroinflammation may have a role in the pathogenesis of psychiatric disorders. More generally, the positive correlation between chronic inflammation and psychiatric disease (e.g., major depressive, bipolar, schizophrenic, and obsessive-compulsive disorders) suggests the presence of an underlying inflammatory process affecting the brain [32,33,34]. Pathways that are common to these disorders include microglial activation, proinflammatory cytokines, molecular mimicry, antineuronal autoantibodies, self-reactive T cells and disturbance of the blood–brain barrier [35,36,37,38]. Innate inflammation may be mechanistically linked to the traditional monoaminergic and glutamatergic abnormalities and increased oxidative injury reported in psychiatric illnesses [35,36,37,38]. In this context, immune treatments are emerging as therapeutic options for subgroups of patients with brain disorders that are associated with an inflammatory phenotype. In the same vein, several studies suggested that a state of neuroinflammation exists in PWS. Doorduin et al. [39] suggested the presence of focal neuroinflammation in the hippocampus during psychosis. Thus, we investigated whether a link between schizophrenia and periodontal disease was established.

A cross sectional pilot study was conducted by Fawzi et al. [40] to estimate the prevalence and quantity of *Porphyromonas gingivalis* in saliva of schizophrenia patients compared to that of nonpsychiatric controls. They demonstrated a significantly higher prevalence and quantity of *P. gingivalis* in saliva of PWS compared to that of controls. Moreover, they found a positive correlation between quantity of *Porphyromonas gingivalis* cells and severity of psychopathology of schizophrenia. In the same vein, Shetty et al. [18] sought to explore through a cross-sectional study the possible bidirectional link between schizophrenia and the periodontal disease. In this study, the periodontal status of 250 PWS was assessed by examining the following 3 parameters: Gingival Index (GI), Plaque Index (PI), and Probe Pocket Depth (PPD). All patients with antipsychotic treatment had no history of systemic disease or periodontal treatment in the past. The results were analyzed according to the duration of schizophrenia, the highest mean values of GI, PI and PPD were found in the group of PWS diagnosed for 11 years and more, followed by the 1–3-year group, the 4–6-year group, and the 7–10-year group. The differences in means between each group were significant, and they found a significant difference between 11 years and above group and all the other groups (*p* < 0.001). The authors concluded that schizophrenic patients have a higher risk of developing periodontal disease and that this risk may be increased by the drugs [41].

### 3.5. Impact of Drugs Such as Valproate on the Microbiome and Schizophrenia

Yolken et al. [19] found that the presence of phiadh was significantly associated with valproate administration. Valproate is commonly used as a mood stabilizer. But Yolken et al. [19] did not find lactobacillus phage in the 6 patients in this study taking valproate, whereas phage was found in 17 of 35 individuals not taking valproate (χ^2^ = 4.98, *p* = 0.026). 

In another work [42], the involvement of oxidative stress in psychiatric pathophysiology including schizophrenia was studied. They did not discuss the microbiome, but they provided animal data on the antioxidant properties of antipsychotics, such as olanzapine, risperidone, or clozapine (contrary to the other antipsychotics such as haloperidol or chlorpromazine, which are pro-oxidants). They also speak about antioxidant properties of mood stabilizers such valproate or lithium, which prevent or reverse lipid peroxidation. Those are issues that could confuse the link between schizophrenia and (oral) microbiome, because of the importance of the oxidative stress (better known in GBA). Studies about oxidative stress in GBA [43] suggest a research focus for OBA.

It is interesting to pinpoint that the research team of Shangaï did a metabolomic work [23] about oxidative stress in schizophrenia. This method is a promising approach which depicts the pathophysioplogical mechanisms of complex diseases or disorders. The authors of this paper discovered two dysregulated metabolic pathways in schizophrenia: an upregulation arachidonic acid-related (a component of the oxidative stress and inflammatory metabolic network) pathway and a downregulation aromatic amino acid-related pathway. Furthermore, the carnitine is an antioxidant lipid which was identified as a promising diagnostic biomarker for schizophrenia with an area under the curve of 0.997 in this study.

## 4. Discussion

The aims of this work were to examine the impact of the oropharyngeal microbiome in the pathophysiology of schizophrenia and to clarify whether there might be a bidirectional link between the oral microbiota and the brain in a context of dysbiosis-related neuroinflammation. In the articles studied, we observed significant differences between the oral microbiota of schizophrenic patients and controls. *Lactobacillus phiadh* was found to be more abundant in schizophrenic patients, similarly, a greater abundance of lactic acid bacteria, in particular *Lactobacillus gasseri*, was found [19] while the levels of *Neisseria subflava*, *Weeksellaceae*, and *Prevotella* were significantly lower [20]. In contrast, streptococcal levels were higher in PWS cases [24]. A significantly higher prevalence and amount of *Porphyromonas gingivalis* was found in the saliva of PWS compared to that of controls [40]. *Porphyromonas gingivalis* could lead to a state of neuroinflammation [30,31]. Finally, there is also a disturbance in the metabolic functions of saliva [25].

The study conducted by Yolken et al. [19] found an increase of *lactobacillus* in samples from schizophrenic patients compared to controls. The primary host bacteria for *Lactobacillus phage phiadh* is *Lactobacillus gasseri*, a common component of the oral and gastrointestinal mucosae and is capable of binding to intestinal epithelium. *L. gasseri* elicits various health benefits through its antimicrobial activity, bacteriocin production, and immunomodulation of the innate and adaptive systems [18], this bacterium is therefore currently used as a probiotic [41]. For Yolken et al. [19], *Lactobacillus gasseri* correlated moderately with the levels of *Lactobacillus phage phiadh*, and this suggests that in many cases the phage infection is in a lysogenic state. Reactivation of the virus can be induced by changes in environmental conditions, leading to the killing of the host bacteria. Moreover, *Lactobacillus phage* may have other impact on the ecology of bacteria by the control of additional species of *Lactobacilli,* and perhaps other bacteria by mediate plasmid transfer. Some phages will be able to modulate the immune system independent from their ability to modulate the level of bacteria, but it is not known if *Lactobacillus phage phiadh* has these properties.

It was found that the host of this phage, *Lactobacillus gasseri* is at least 400 times more abundant in PWS than controls [20,44]. As pointed out by Nguyen et al. [21] this observation is surprising given that this bacterium used as a probiotic can boost immunity and has benefits, whereas schizophrenia was associated with chronic inflammatory states. However, this increase in *lactobacilli* has not only been demonstrated in the oropharynx of schizophrenic patients. Indeed, an increase of *lactobacilli* was also found in fecal microbiota in patients with first episode psychosis [45].

A recent study on the management of obesity [46] using the gut microbiota of mouse models showed that a combination of B. animalis subsp. lactis with Lactobacillus gasseri significantly limited the body weight gain and increase in body fat induced by a high-fat diet (HFD) (*p* < 0.05). This association also significantly decreased the HFD-induced increase in inflammatory gene expression (*p* < 0.05 or 0.01). The impact of these probiotic strains should be studied at the level of the oral microbiota.

Yolken et al. [19] found that the presence of phiadh was significantly associated with valproate administration. De Theije et al. [47] investigated the microbiota composition in a murine model of autism spectrum disorders. The mice were exposed in utero to valproic acid. This prenatal exposition seemed to have an impact on operational taxonomic units (which are similar to the concept of species) assigned to certain genera of *Bacteroidetes* and *Firmicutes*, which could impact the inflammation process. Other treatments (such antipsychotics) could made confusion because of antioxidant properties. The role of oxidative stress is better known in the GBA and could be an interesting research focus.

Hashioka et al. [22] noted that the link is not yet proven between oral microbiome and schizophrenia. However, this is an interesting line of research, as this link was studied more in the case of Alzheimer’s disease and depression. Dioguardi et al. [48] have studied the role of periodontitis and periodontal bacteria in the onset and progression of Alzheimer’s disease. They highlighted that the bacterial load and the inflammatory process associated with periodontal disease can lead to a state of neuroinflammation favoring the onset of the disease, a statistically significant relationship between IgG, *Porphyromonas gingivalis* and dementia was found, suggesting a correlation between periodontitis and neurodegenerative disease.

Qing Y et al. [25] found a role for saliva in the initiation of schizophrenia by suggesting a synergy with *Firmicutes* and *Actinobacteria*, *Fusobacteria,* and *Acidobacteria*. The ratio of *Firmicutes* to *Proteobacteria* found in schizophrenia also exists in primary Sjögren’s syndrome. Schizophrenia and Sjögren’s syndrome share this inflammatory aspect (Sjögren’s syndrome notably involves the salivary glands). This ratio may be important in understanding the low-grade inflammation response. The study of saliva components [25] showed that saliva from schizophrenia patients shared similar microorganisms. They also showed that other authors focused on the blood microbiome (which results from translocation from the oral cavity and gut) and also found this increased alpha diversity (for PWS) Qing et al. [25] noted that the discrepancies (between the three niches: oral, gut, and blood) in the differences in microbial alpha-diversity would be reasonable. 

Other authors [48] found this increased alpha-diversity for PWS in blood microbiome with a transcriptome (RNA) analysis, whereas they cannot know the origin of these bacterial communities. A case-control study [49] made a gut microbiota profiling for PWS and indicated a close association between oral and gut microbiota in schizophrenia, higher alpha and beta diversities for PWS than for that of healthy controls. This article also suggests that oral resident bacteria (Lactobacillus oris, Streptococcus salivarius) may colonize the gut in schizophrenia patients, leading to a significant enrichment of oral cavity resident bacteria. The authors added that it could be explain by a decrease immune surveillance in the gut. This point gives us another hypothesis for the OBA: the link between oral microbiome and schizophrenia could be made throughout the gut microbiota [50]. 

A case-control study called “metagenome-wide association study” [51] reports the presence of bacteria that are often present in the oral cavity (*Veillonella atypica*, *Veillonella dispar*, *Bifidobacterium dentium*, *Dialister invisus*, *Lactobacillus oris*, and *Streptococcus salivarius)* were more abundant in PWS than in healthy controls, indicating an association between oral and gut microbiota in schizophrenia. Furthermore, the authors constructed a metagenomic operational taxonomic unit network to depict the co-occurrence correlation between the schizophrenia-associated gut bacteria, and they discovered that the majority of the species originated from the oral cavity. They added that this pointing to the relation between oral resident bacteria and gut bacteria, suggesting that oral resident bacteria in a synergistic manner may colonize the gut in schizophrenic patients. A study is currently underway to clarify the relationship between the composition of the oral and intestinal microbiota in patients with psychosis or schizophrenia and healthy controls [50].

## 5. Perspectives

Despite considerable efforts to characterize, diagnose, and find a promising treatment for schizophrenia, efforts are still needed to understand the reason and mechanism of this disease. From this perspective, for the treatment of psychotic disorders, the continuous use of pro- and prebiotics is generally suggested to prevent alteration of the gut microbiota and enhance natural immunity [52] with contrasting results, especially in schizophrenia [53]. Studies are underway to identify the most effective microbial strains and the appropriate dose of fiber/prebiotics for their proliferation with therapeutic potential [54,55]. Regarding periodontogenic probiotic strains, they could facilitate oral hygiene measures in patients with Alzheimer’s disease [56]. From this perspective, therapeutic strategies aimed at modulating oral dysbiosis as well as modifying the amyloids produced by bacteria or reducing their production could also represent a future line of research for the treatment of schizophrenia. 

Nutraceuticals are known for their health benefits: reduction of the risk of cancer and heart disease, but also the prevention or treatment of hypertension, hypercholesterolemia, overweight, osteoporosis, diabetes, arthritis, digestive disorders, and constipation, not to mention headaches [57,58,59]. It was shown that nutraceuticals may have therapeutic properties such as anti-inflammatory and antibacterial effects that may be beneficial for the treatment of periodontitis [60]. 

A recent study showed that marine bioactive ingredients such as seaweed extracts, n-3 PUFAs, sea cucumber extracts and marine bacterial metabolites have the ability to inhibit oral pathogens, suppress their biofilms and regulate the cancer cell cycle [61]. This marine bioactive ingredient could play a role in the inhibition of oral pathogenic bacteria, elimination of inflammation and antitumor action. This discovery opens interesting research perspectives for the prevention and stabilization of psychotic disorders through the use of this bioactive ingredient packaged, for example, in the form of chewing gums or sugar-free tablets. 

## 6. Limitation

None of the studies selected in this review established a formal causal link between the oral microbiota and schizophrenia because of a large number of confounding biases such as certain environmental exposures that affect the microbiota: use of drugs such as cannabis, sexually transmitted diseases that can affect the mouth, alcohol, respiratory viruses, anticholinergic treatments, and oral hygiene that is often poor in PWS [62]. Fawzi et al. [40] noted that detection of *Porphyromonas gingivalis* was correlated with being less educated. There was also a significant correlation between the detection of *Porphyromonas gingivalis* and smoking [40]. In the study by Castro–Nallar et al. [20], all controls were nonsmokers. In this case, it is possible that the effects of smoking are confused with those of schizophrenia. Decreased abundance of *Neisseria* and *Capnocytophaga* was associated with smoking [26]. But it is difficult to make a link with mental illness.

## 7. Conclusions

In this study, we pointed out the presence of phageoma in patients suffering from schizophrenia and that periodontal disease reinforces the role of inflammation in the pathophysiology of schizophrenia. Moreover, saliva could be an interesting substrate to characterize the different stages of schizophrenia. However, we have only few studies at our disposal on the subject, which are of limited scope and are produced by the same research group. At this stage of knowledge, it is difficult to conclude on the existence of a bidirectional link between the brain and the oral microbiome. Further studies are still needed to clarify the link between the oral microbiome and schizophrenia.

## Figures and Tables

**Table 1:** Analysis of selected articles.

Reference	Study Design	Objectives	Mains Results	Limitations
Shetty S et al., 2014 [18]	Cross-sectional epidemiological study 250 patients (140 males and 110 females) with a positive history of schizophrenia	To explore the possible bidirectional link between periodontal disease and schizophrenia.	The study shows that patients who were schizophrenic for a longer duration of time (*p* < 0.001) have increased evidence of poor periodontal condition, demonstrated by gingival and plaque indexes. The findings suggest that the role of periodontal disease in the pathogenesis of schizophrenia cannot be ruled out.	Further long-term interventional studies involving periodontal management of PWS and monitoring the cytokine profile followed by assessment of changes in the schizophrenic status of these patients need to be undertaken.
Yolken RH et al., 2015 [19]	Case control 41 PWS and 33 control individuals without a psychiatric disorder	To analyze to characterize bacteriophage genomes in the oral pharynx of PWS and control individuals without a psychiatric disorder	*LPP*, with PWS, was significantly greater than the number of matches in the controls. The level of *LPP* was correlated with an increased rate of comorbid immunological disorders with *PWS*.	The level of *LPP* was found to be associated with the administration of valproate, commonly used in the treatment of epilepsy or as a mood stabilizer in schizophrenics.
Castro–Nallar E et al., 2015 [20]	Case control 16 individuals with schizophrenia and 16 controls	To characterize the schizophrenia microbiome by interrogating the oropharyngeal microbiome structure regarding its taxonomic and functional diversity.	Lactic acid bacteria were relatively more abundant in schizophrenia including *Lactobacillus* and *Bifidobacterium* with the largest effect found in *Lactobacillus gasseri,* which appeared to be at least 400 times more abundant in *PWS* than in controls. *PWS* showed decreased oral microbial biodiversity and an increased number of metabolic pathways related to metabolite transport systems including siderophores, glutamate, and vitamin B12 in PWS. In contrast, carbohydrate and lipid pathways and energy metabolism were abundant in controls.	The fact that all controls were non-smokers, although statistically accounted for in inferences, might confound the effects of schizophrenia from those of smoking on microbiome composition.
Dickerson F et al., 2017 [5]	Review	To summarize what is known about immune alterations and the microbiome based on human studies in schizophrenia and bipolar disorder.	The part on the oral microbiota takes up the results of the studies by Robert H. Yolken et al. and Eduardo Castro–Nallar et al.	Observational study.
Nguyen TT et al., 2018 [21]	Review	To highlight gaps in our knowledge, potential implications for diagnosis and therapeutic interventions, and outline future directions for microbiome research in psychiatry.	*PWS* showed decreased oral microbial biodiversity. At the genus level, lactic acid bacteria (*Lactobacillus* and *Bifidobacterium*) were relatively more abundant in schizophrenia, particularly species *Lactobacillus gasseri*. Notably, lactic acid bacteria are often considered health-promoting and anti-inflammatory. *LPP* in *PWS*, which is a bacteriophage that preferentially infects the bacteria *Lactobacillus gasseri.* This study also observed a positive association between colonization with *LPP* and comorbid immunological diseases. These conflicting results may be due to differences between populations, as schizophrenia is a very heterogeneous disease, or underscore the complexity of community relationships within the microbiota.	Observational study.
Hashioka S et al., 2019 [22]	Review	To focus on the biological and epidemiological evidence of possible causal links of periodontitis to the selected neuropsychiatric disorders, namely Alzheimer, Depression, Schizophrenia, Ischemia, Stroke, Parkinson	The study showed that evidence of a significant relationship between periodontitis and schizophrenia is not yet accumulated. Only one cross-sectional study with a small sample size concluded that patients with schizophrenia have a high risk of periodontitis and there is an even higher risk in those who are taking antipsychotics that reduce salivary secretion and cause xerostomia.	A small sample size with schizophrenia. This review article focuses on possible causal links of periodontitis to schizophrenia but other neuropsychiatric disorders.
Cui G et al., 2020 [23]	Case control 54 with metabolic profile of schizophrenia 54 healthy controls	To characterize metabolites in the peripheral circulation to deepen our understanding of the pathogenesis of schizophrenia (using Fourier transform-ion cyclotron resonance-mass spectrometry (FT-ICR-MS))	The authors discovered two dysregulated metabolic pathways in schizophrenia: an upregulation arachidonic acid-related pathway, and a downregulation aromatic amino acid-related pathway. The carnitine was identified as a promising diagnostic biomarker for schizophrenia with an area under the curve of 0.997. An imbalance of the redox homeostasis in schizophrenia	Low level of evidence
Yolken R et al.,2021 [24]	Case control 316 individuals, including 121 PWS, 62 with mania, 48 with major depressive disorder, and 85 controls without a psychiatric disorder	To confirm the link between *PWS* and altered oropharyngeal microbiome with a larger sample of *PWS*.	The study showed that the oropharyngeal microflora of *PWS* and individuals with mania differed from controls. Three of the taxa, *Neisseria subflava*, *Weeksellaceae*, and *Prevotella*, were decreased in *PWS* or mania as compared to that of controls, while *Streptococci* wereincreased in these groups. *Neisseria subflava* was also positively associated with cognitive functioning. Altered beta-diversity in *PWS* and mania as compared to control individuals without a psychiatric disorder.	There are a number of environmental exposures which might be increased in individuals with psychiatric disorders, and which were not directly measured in this study. Inhaled drugs such as cannabis, increased exposure to sexually transmitted diseases, to respiratory viruses, to effects of alcohol and to medications such as anticholinergic agents. Patients with inadequate periodontal care may not have detailed dietary. This study was cross-sectional and did not measure changes over time
Qing Y et al., 2021 [25]	Case control 85 patients with first episode schizophrenia (FES) 43 with clinical high risk (CHR) 80 healthy controls (HCs)	To investigate the salivary microbiome in the context of schizophrenia. To characterize the microbial profiles at different clinical stages of the schizophrenia. To gain understanding of the function of salivary microbes in the initiation of schizophrenia.	The authors found a high ratio *Firmicutes/Proteobacteria* in PWS in the salivary microbiome. They added that H2S-producing bacteria could be potential diagnostic biomarkers for FES and CHR and precede the onset of the trouble. The study showed that the metabolic functions of the salivary microbiome were disturbed in schizophrenia.	It is an observational study. It cannot prove the causal relationship between the salivary microbiome and schizophrenia. Lack of metagenomic data to determine the actual microbial gene content in the salivary microbiome. The dental status and oral hygiene not sufficiently considered

LPP: Lactobacillus phage phiadh; PWS: Persons with schizophrenia.

## Data Availability

Not applicable.
